# Innovative Mode of Logistics Management of “Internet of Things + Blockchain”-Integrated E-Commerce Platform

**DOI:** 10.1155/2022/7766228

**Published:** 2022-05-29

**Authors:** Hou Yujie, Hao Qiuxia

**Affiliations:** School of International Business, Qingdao Huanghai University, Qingdao, Shandong 7266000, China

## Abstract

The innovation of logistics management mode plays a great role in promoting the operation timeliness of an e-commerce platform. The question of how to realize the research on the innovation mode of logistics management of the e-commerce platform with the integration of “Internet of things + blockchain” is the current development trend. Based on this, this paper studies the influencing factors of logistics management of an e-commerce platform under the discrete analysis strategy of “Internet of things + blockchain.” First, the logistics management analysis model of an e-commerce platform integrating “Internet of things + blockchain” is proposed. The dynamic correlation function is used to simulate the logistics information in the e-commerce platform. Through the extreme value of the dynamic correlation function curve in the detection process, the operation signal is restored, and then its logistics management is analyzed. Second, the influencing factors in the logistics management of the e-commerce platform are analyzed, comprehensive analysis is done by using the “Internet of things + blockchain” integration model, the dynamic logistics information in the operation of the e-commerce platform is accurately gathered, and the multidimensional hierarchical method is used for quality evaluation. Finally, the effectiveness of the logistics management analysis model of the e-commerce platform is verified by many experiments.

## 1. Introduction

With the rapid development of the platform, the logistics management system has been greatly improved and gradually developed in the direction of informatization, networking, integration, and automation. This makes the trading activities break the restrictions of time and space and greatly improve the transportation efficiency. E-commerce and logistics management, the so-called e-commerce, generally speaking, refer to business transactions on the network.

Logistics management refers to the use of scientific management methods to coordinate and control logistics activities in a planned and organized way. So that all logistics activities can be carried out smoothly, so as to reduce logistics cost and improve logistics efficiency. The modern logistics management technology is developed on the basis of e-commerce, and there is a very close relationship between them. Logistics management based on e-commerce consumers can directly order the services and products of some enterprises through the platform provided by e-commerce. Then, according to the information provided by customers, the information flow is transmitted to the enterprise. Then, the goods are delivered to consumers through logistics through the material management department. These are the information provided by the e-commerce platform, which not only ensure the development of logistics but also improve consumers' satisfaction with products and services.

China has certain data advantages in the research of logistics management of e-commerce platform, and there are many simulation research methods, including composite logistics simulation, differentiated local simulation, and multiparameter simulation [1]. In addition, in the process of studying the impact of intelligent network on the logistics management of the e-commerce platform, one of the hot spots is the combination of “Internet of things + blockchain.” From the analysis of the impact of the e-commerce platform on efficiency, in addition to the impact on customers' e-commerce platform usage habits, it also involves the logistics management part [2]. At the beginning of the 21st century, quantitative analysis methods began to appear in the research on the logistics management of e-commerce platform and the quantitative analysis methods continued to increase. Since then, the theory of the impact of “Internet of things + blockchain” on the logistics management of e-commerce platform began to pursue a diversified balance [3]. Internationally, the most critical simulation part affecting efficiency lies in the evaluation of the simulation part of the e-commerce platform, and the efficiency of logistics management of the e-commerce platform is improved through corresponding simulation [4]. In this context, based on the improved “Internet of things + blockchain” integration method, this paper puts forward the impact analysis model of logistics management of the e-commerce platform.

This paper studies the logistics management evaluation model of the e-commerce platform integrating “Internet of things + blockchain,” which is mainly divided into four chapters. Chapter 1 introduces the research background and research objectives. Chapter 2 introduces the research status of the impact of “Internet of things + blockchain” on the logistics management of the e-commerce platform. Chapter 3 constructs the logistics management evaluation model of the e-commerce platform integrating “Internet of things + blockchain,” and adopts the multidimensional composite “Internet of things + blockchain” integration under the multidomain. In Chapter 4, the logistics management impact simulation system of the “Internet of things + blockchain”-integrated e-commerce platform constructed in this paper is tested, and the experimental results are analyzed to draw a conclusion.

Compared with the existing research methods (such as the research method of random construction of sampling data), the innovation of this paper is to propose a discrete analysis system based on the improved “Internet of things + blockchain” fusion. The system can not only realize the daily recording and storage of information and data contained in the operation process of different “Internet of things + blockchain” e-commerce platforms but also make full use of the regional differences in the logistics management process of each e-commerce platform through data comparison and analysis and realize the closed-loop evaluation of logistics management of the e-commerce platform in the process of “Internet of things + blockchain.”

## 2. Related Work

The Internet of things technology has developed rapidly in recent years, and there are more and more application scenarios. With the development needs of the Internet of things technology, blockchain technology also came into being, realizing decentralized digital applications. Relevant domestic logistics engineering and management research institutes have also carried out research on the combination of the Internet of things technology and blockchain technology [5]. Jiang and other scholars summarized and analyzed according to the operation law of the logistics platform and found that there is a problem of low intelligence in the influencing factors of e-commerce platforms on logistics management [6]. Sun and other scholars improved the discrete representation method of e-commerce platforms in the operation process according to the different characteristics and types of logistics information in different regions [7]. Dong and other scholars conducted differentiation analysis according to the process of logistics management of the e-commerce platform and realized the specific representation of the logistics management characteristics of the e-commerce platform according to the fixed key information [8]. Li and other scholars used methods different from conventional ideas to realize the vectorization processing of e-commerce platform signals and used different modes to formulate theoretical evaluation standards and data consistency rules, and studied the operation laws of different types of logistics platforms [9]. De Ku and other scholars summarized the logistics management analysis methods of e-commerce platforms at this stage, classified them according to their identification principles, and analyzed the advantages and disadvantages of different types of e-commerce platforms in the process of logistics management analysis [10]. Gunasekaran A and other scholars have identified the existing logistics management analysis methods of e-commerce platforms with ultrahigh discrimination according to the data information of different types of e-commerce platforms and achieved high accuracy analysis from the aspects of their composition and operation mode [11].

Based on the above research status, it can be seen that there is a lack of research on logistics management analysis of the e-commerce platform combined with intelligent network, and there are few research results [12]. Therefore, although domestic and foreign researchers have done a lot of basic research in the logistics management of the e-commerce platform, the innovation in the research results is relatively insufficient [13]. In addition, in the current research on the innovative mode of logistics management of e-commerce platform, most of them adopt the joint analysis method of simulated data combined with some real data [14]. On the other hand, in the infiltration tool evaluation and optimization of logistics management of the e-commerce platform, the efficiency of the discrete evaluation method adopted is not high, and its internal relevance is weak, resulting in the low transferability of most evaluation models [15]. Therefore, it is of great significance to study the logistics management and discrete evaluation analysis method of the e-commerce platform integrating “Internet of things + blockchain.”

## 3. Methodology

### 3.1. Application Idea of “Internet of Things + Blockchain” Integration in the Discrete Analysis of Logistics Management of an E-Commerce Platform

**Figure 1:**
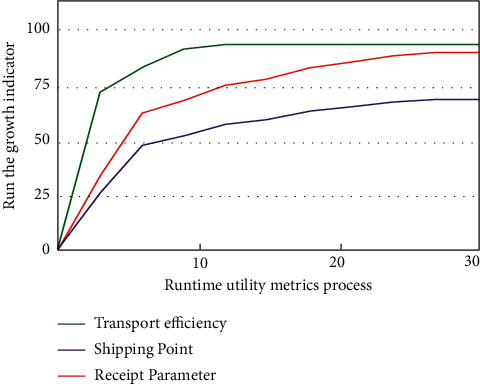
Common evaluation indicators of logistics efficiency.

The Internet of things technology is an intelligent technology that combines hardware and software to realize the highly coordinated application of data and resources through interconnection. The Internet of things technology has been applied in different scenarios. Especially in the logistics management process of e-commerce, the comprehensive, coordinated, and unified allocation of logistics data can be realized through the Internet of things technology. In the process of data collection and analysis, the commonly used methods of Internet of things technology are neural network algorithm, mountain climbing algorithm, simulated annealing algorithm, and “Internet of things + blockchain” fusion algorithm [16]. At present, a variety of algorithms have been well applied in many engineering fields such as production scheduling, control engineering, computer vision, neural network, image processing, and accurate query of database information [17]. In addition, different types of optimization methods have emerged in the logistics management of e-commerce platform data, especially in the process of analyzing the logistics management strategy of the e-commerce platform. Among many methods for quantitative analysis of logistics management results of the e-commerce platform, “Internet of things + blockchain” integration has also attracted attention in recent years. This discrete method, combined with a mathematical method and information method, was applied to solve the problems of information systems in the early stage. Therefore, in the process of discrete data analysis and processing, mathematical modeling through the integration of “Internet of things + blockchain” technology is to determine the mathematical relationship between many factors of a gray system. In the process of logistics management analysis of the e-commerce platform, the feature extraction of discrete data is used to distinguish the differences of logistics management between different signals according to the extraction results. By using the blockchain technology, we can realize the high-intensity unified deployment application and information analysis of logistics signal data. Compared with the conventional logistics management analysis method of e-commerce platforms, this signal processing process has significantly improved the accuracy and stability of seepage tool evaluation. In the process of logistics management analysis of e-commerce platforms, the common analysis curve is shown in Figure 1.

### 3.2. Construction Process of the Logistics Management Analysis Model of an “Internet of Things + Blockchain” E-Commerce Platform

**Figure 2:**
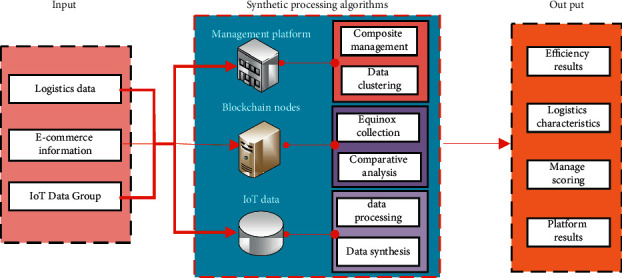
Composite synthesis processing algorithm processing process.

The signal processing process of e-commerce platforms by the neural network algorithm is shown in Figure 1. With the deepening of the research on discrete data processing of “Internet of things + blockchain” fusion by scholars, these can be analyzed and processed by using the “Internet of things + blockchain” fusion theory. At the same time, it is also necessary to obtain the key features in the signal through blockchain. The process of the composite synthesis processing algorithm shown in Figure 2.

Building the logistics management analysis model of e-commerce platform requires three steps. The first step is to measure the degree of correlation according to the relationship between factors in the Internet of things nodes. Its basic idea is to sort according to the degree of correlation. In the application, the original data matrix is initialized, and then, the reference data matrix is formulated. The e-commerce platform signal *E*_*m*_(*t*) can be marked as follows:

(1) $$\begin{array}{c} E_{m}\left(t\right)={\left[\frac{E_{1}\left(t\right)+E_{2}\left(t\right)+\text{…}+E_{\text{n}}\left(t\right)}{n}\right]}^{2}. \end{array}$$

The second step is to calculate the absolute difference between the random subfactor and the main factor. The formula runs as follows:

(2) $$\begin{array}{c} \Delta_{m}\left(r,\text{t}\right)=w_{m}^{2}\left(r\right)-w_{\text{n}}^{2}\left(\text{t}\right),\\ \Delta_{\text{n}}\left(r,\text{t}\right)=w_{n}^{2}\left(r\right)+w_{m}^{2}\left(t\right), \end{array}$$

where *m* and *n* are two-dimensional neural layers, *r* represents the reference data column, and *t* represents the individuals in different data columns. The third step is to calculate the correlation degree *ο*_m_(*t*) between the subfactor and the main factor. The formula is as follows:

(3) $$\begin{array}{c} {\left|{ο}_{\text{m}}\left(t\right)\right|}^{2}=\frac{\left|w_{t}^{2}\left(r\right)-w_{t}^{2}\left(t\right)\right|}{\sqrt{n}}. \end{array}$$

### 3.3. Quantitative Evaluation Process of the Logistics Management Analysis Model of an “Internet of Things + Blockchain” E-Commerce Platform

The quantitative evaluation of the logistics management analysis model of e-commerce platforms is a complex process [18]. This paper analyzes an Internet of things data management system based on the blockchain and shared environment. The system includes data acquisition and storage module, which are used to obtain the Internet of things data and partition the data. The data slice is obtained and stored in the regional server. The hash address corresponding to the data slice is stored on the blockchain, and the smart contract module is used to receive the data transaction request sent by the data consumer and the corresponding hash address. The corresponding data slice is obtained according to the hash address and the transaction is executed.

In order to further study the “Internet of things + blockchain,” in the multidimensional hierarchical method using the integration of “Internet of things + blockchain,” first the problems are clarified, the system objectives are determined, and the scope of decision-making problems is analyzed, which are generally carried out in the following steps.

The first step is to establish an analytic hierarchy process (AHP) system. Combined with the logistics management research theory of the e-commerce platform and time domain signal analysis, a discrete analysis is carried out in the comprehensive change process of the simulation signal of logistics management of e-commerce platforms, combined with the “Internet of things + blockchain” discrete data simulation system based on an ant colony recursion theory. The feature extraction algorithm is applied to the simulation model of “Internet of things + blockchain,” and the logistics management analysis method of the “Internet of things + blockchain” e-commerce platform is established based on the feature extraction algorithm. This link is divided into data collection, data processing, result feedback, and so on. *M*(*t*) indicates the feasibility of analytic hierarchy process.

(4) $$\begin{array}{c} M\left(t\right)=\frac{c\left(t\right)-p\left(t\right)+1}{c\left(t\right)+p\left(t\right)}, \end{array}$$

*c*(*t*) and *p*(*t*) are hierarchical functions. When the corresponding value of critical feasibility is reached, it is recognized as the effective value.

The second step is to determine the analysis indicators according to different logistics management, and then divide the system into different levels. In order to facilitate the calculation, the fusion formula method is adopted. This level division reflects the subordinate relationship of each level, but the importance of each index will not be the same.

(5) $$\begin{array}{c} m\left(t\right)=\frac{\sqrt{h{\left(S_{k}\right)}^{2}+n^{2}}}{n}, \end{array}$$

where *b* and *c* are matrix factors. It is followed by judging whether the model has passed the separation test and whether it can be recognized as the effective value only after meeting the separation test requirements.

The third step is to judge the maximum eigenvalue corresponding to the characteristic matrix in the model *λ*. The relative importance ranking can be reflected by normalization. Although this structure can reduce the interference of other factors and objectively reflect the influence of differences, there must be a certain degree of separation correlation in the comprehensive comparison. If the same result is produced, the discriminant function *e*(*h*) should meet the following conditions:

(6) $$\begin{array}{c} \text{e}\left(h\right)={\left(\frac{bw\left(t\right)+cw\left(t\right)}{i}\right)}^{2}+b\text{c}w\left(t\right), \end{array}$$

where *b* and *c* are matrix factors. This is followed by judging whether the model has passed the consistency test and can be recognized as the effective value only after meeting the consistency test requirements.

Using the common reduction mechanism and introducing *k* for simplification and reconciliation, it can be simplified as follows:

(7) $$\begin{array}{c} \text{e}\left(h\right)=k{\left(\frac{bw\left(t\right)+cw\left(t\right)}{i}\right)}^{2}. \end{array}$$

The fourth step is to conduct consistency inspection. The current scaling method is mainly 9-scale method, and its test function RF(t) running formula is as follows:

(8) $$\begin{array}{c} \text{RF}\left(t\right)=\frac{t^{2}+3t+1}{t^{2}+7+t^{-1}+t^{-2}}. \end{array}$$

Finally, we need to improve the above “Internet of things + blockchain” integration and e-commerce platform intervention conditions. In this paper, we mainly improve the blockchain algorithm and use the omega method. This method is simpler to operate. It is only calculated according to the example of importance through the composite comparative analysis. This algorithm has other defects. For example, the business rules and simulation process of e-commerce platforms will directly affect the establishment of the judgment matrix. Affected by subjective factors, it is very prone to objective errors. Therefore, the multidimensional hierarchical method cannot objectively reflect the research problems. It also needs to be evaluated by the fuzzy hierarchical method of “Internet of things + blockchain.”

### 3.4. Design of the Discrete Analysis Model for Logistics Management of an E-Commerce Platform Integrating “Internet of Things + Blockchain”

Under the intervention of multilevel coupling factors, in order to study the impact of different discrete data on the logistics management model of e-commerce platforms, the accuracy of simulation is quantitatively analyzed, but in many cases, there is no quantitative data, so it is necessary to quantitatively transform the qualitative data in the simulation process. [19]. In this case, the commonly used functions include a discrete error analysis function, combined covariant function, and so on [20, 21]. The method used in the standard is a comprehensive evaluation function. The results show that this model can effectively reduce the characteristic value of error parameters, improve the accuracy evaluation and cooperation efficiency of logistics management factors of e-commerce platform, and is suitable for different “Internet of things + blockchain,” technologies and can also adapt to subjective interference signals in the simulation process of “Internet of things + blockchain” [22].

**Figure 3:**
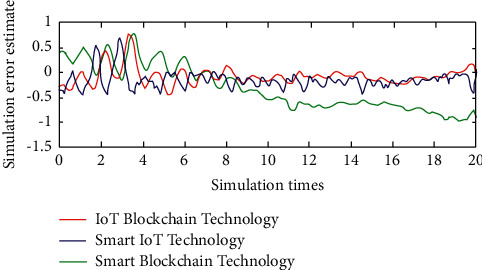
Simulation error estimate.

In the evaluation link of the impact of “Internet of things + blockchain” on the logistics management of human e-commerce platforms, the meaning of data is different, so it is not equivalent to analysis. The original data need to be processed in a dimensionless way, and the proportional transfer method is used in this paper [23]. At present, the behavior decision of IOT terminals depends on the control of the central cloud platform (or edge cloud platform), and its data processing and M2M interaction ability is weak. It can be combined with blockchain, tee (trusted execution environment), and other technologies, which are expected to enhance the intelligence of IOT terminals and make the IOT network have differential distributed intelligence. Blockchain ensures the terminal's identity can be verified and the credibility, reliability, openness, and transparency of data records. If combined with the terminal's hardware computing platform, the terminal can be built into an intelligent platform to complete some local tasks independently. After the normalization process is completed, the relationship between different “Internet of things + blockchain” simulation series can be calculated, and then, the difference between each factor and the main factor at the same observation point can be calculated. The simulation results are shown in Figure 3.

As can be seen from Figure 3, the logistics management analysis model of e-commerce platforms based on the Internet of things has better stability of simulation results, and its change law is better than that of the other two methods. When comprehensively evaluating the simulation results of the impact of “Internet of things + blockchain” on the logistics management of key customer e-commerce platforms, in most cases, the problem of ranking will be involved. Each evaluation object needs to be ranked first, so it needs to be further compared with the help of gray comprehensive evaluation index.

In order to study the logistics management law of the e-commerce platform under the analysis of Internet of things and blockchain, the hierarchical relationship of gray evaluation index adopted in this article is expressed by set, and its operation formula *κ* is as follows:

(9) $$\begin{array}{c} \kappa \left(x\right)=\sum_{i=1}^{p}\left(\frac{{\text{t}}_{ik}-t_{i}}{i+k}\right). \end{array}$$

After the operation formula of its management law is obtained, it is judged. The discriminant of the integration judgment formula *y*(*t*) and the characteristic judgment formula *z*(*t*) is as follows:

(10) $$\begin{array}{c} y\left(t\right)=\sum_{\text{i}=1}^{n}\left(\frac{t_{ki}}{k+i}+\frac{t_{kj}}{k+j}\right),\\ z\left(t\right)=\frac{\sum_{i=1}^{n}\left(t_{ki}/k+i\right)}{\sum_{i=1}^{n}\left(t_{ki}/k-i\right)}. \end{array}$$

In the logistics management evaluation function of this e-commerce platform, each secondary indicator will also have its own weight coefficient, which is expressed by different coefficients. The weight vector function of each layer can be classified as *u*_1_(*t*) or *u*_2_(*t*). The operation formula is as follows:

(11) $$\begin{array}{c} u_{1}\left(t\right)=\frac{2t^{5}+\sqrt{t^{2}+7+}t^{-1}}{3\sqrt{t^{2}+1}},\\ u_{2}\left(t\right)=\frac{2t^{5}-\sqrt{t^{2}+7+}t^{-1}}{3\sqrt{t^{2}+1}}, \end{array}$$

where *t* is discrete data.

The following discriminant is used to judge the classification:

(12) $$\begin{array}{c} P\left(t\right)=\sum_{i=1}^{n}\frac{y\left(t\right)}{z\left(t\right)}. \end{array}$$

If *P*(*t*) is greater than 1, it is classified as *u*_1_(*t*); otherwise, it is classified as *u*_2_(*t*).

## 4. Result Analysis and Discussion

### 4.1. Evaluation Index of the Discrete Analysis Experiment of Logistics Management of an E-Commerce Platform Integrating “Internet of Things + Blockchain”

**Figure 4:**
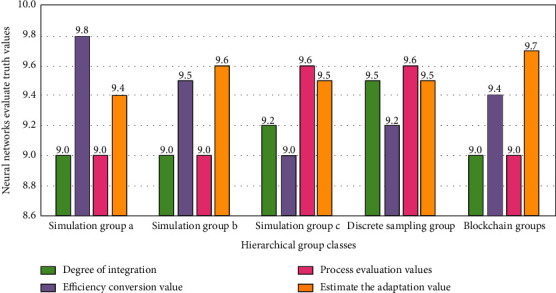
Simulation versus actual evaluation.

In this study, the simulation evaluation results of the influencing factors of logistics management of the e-commerce platform under different blockchain nodes are divided into a significant impact and no impact. After determining the classification levels, these levels are assigned and scored, and the ten point assignment method is used to evaluate their integration degree, efficiency conversion value, process evaluation value, and estimated adaptation value. The evaluation results are shown in Figure 4.

### 4.2. Experimental Results and Analysis

**Figure 5:**
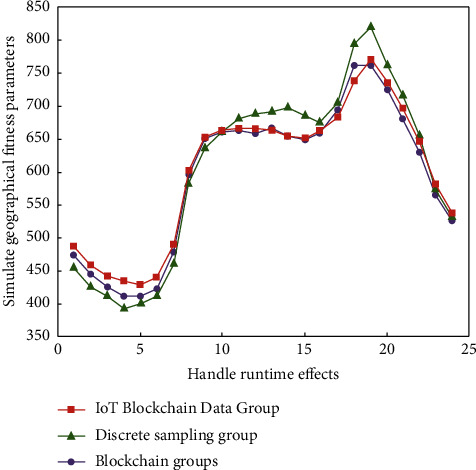
Logistics management analysis of e-commerce platforms.

Figure 5 shows the experimental results of logistics management analysis of three groups of e-commerce platforms under the optimization of “Internet of things + blockchain.” MATLAB software can effectively process the relevant data in this evaluation model.

**Figure 6:**
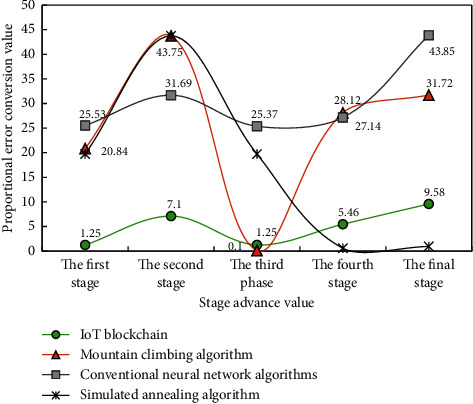
Scale error variation law diagram.

Figure 6 shows the variation law of proportional error in the discrete analysis of logistics management of different e-commerce platforms by four collection and processing methods under different analysis completion degrees.

As can be seen from Figure 6, with the increase of analysis completion, the accuracy of discrete dynamic analysis corresponding to the four collection and processing methods is different, but presents a similar change law. When the analysis completion degree is the same, the analysis stability and accuracy corresponding to the “Internet of things + blockchain” fusion are the highest, followed by the neural network algorithm. When the completion degree of the four collection and processing methods is 70%, the corresponding analysis accuracy is the highest because the effectiveness of the corresponding cycle analysis times is the highest in the process of discrete dynamic analysis of experimental data.

**Figure 7:**
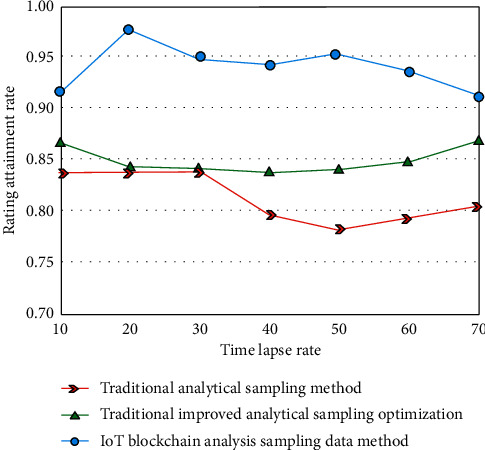
Comprehensive scoring of the logistics management platform.

For example, the characterization of the current data is classified into different types of evaluation criteria, and the evaluation results are refined according to the different characterization criteria of the current e-commerce data [24], Finally, the preliminary evaluation and secondary evaluation are carried out according to the known industry reference standards. In this way, the evaluation and analysis of the experimental results can be well completed, which is consistent with the current mainstream evaluation rules and more persuasive and then calculate the weight and score. The experimental simulation analysis results are shown in Figure 7.

As can be seen from Figure 7, the results of the “Internet of things + blockchain” model are more stable with the increase of analysis times and are better than the experimental analysis results of other methods, which shows that compared with the traditional neural network e-commerce platform analysis model and its optimization algorithm, the logistics management analysis model of e-commerce platforms analyzed by the “Internet of things + blockchain” model has a better analysis effect in the logistics management analysis of local e-commerce platform signals. Finally, through model analysis and calculation, it can be concluded that the efficiency improvement rate of this method can reach more than 90, which is better than other collection and analysis methods.

## 5. Conclusion

The current e-commerce platform has some problems in logistics management, such as less quantitative statistics and large error. Based on this, this paper studies the logistics management analysis model of e-commerce platforms based on the integration and analysis strategy of “Internet of things + blockchain.” First, it summarizes the current situation of logistics management analysis of the e-commerce platform and puts forward ideas for improvement and optimization according to its existing problems. Second, through the differences of different types of e-commerce platforms in logistics management, we extract their features and formulate classification strategies. Third, it analyzes the influencing factors of logistics management of the e-commerce platform, makes a comprehensive analysis by using a multidimensional data model, studies the influencing factors involved, and summarizes their influence law and weight ranking in the operation process; Finally, the logistics management analysis model of e-commerce platforms is verified by experiments. The results show that compared with the conventional methods, the logistics management analysis model of e-commerce platforms proposed in this study has a better reliability and lower error rate. However, this study only considers the processing of simulation data, not the elimination of real multidimensional interference signals, so it can be further studied.

## Data Availability

The data used to support the findings of this study are available from the corresponding author upon request.
